# Astaxanthin, a Carotenoid, Stimulates Immune Responses by Enhancing IFN-γ and IL-2 Secretion in Primary Cultured Lymphocytes *in Vitro* and *ex Vivo*

**DOI:** 10.3390/ijms17010044

**Published:** 2015-12-29

**Authors:** Kuan-Hung Lin, Kao-Chang Lin, Wan-Jung Lu, Philip-Aloysius Thomas, Thanasekaran Jayakumar, Joen-Rong Sheu

**Affiliations:** 1 Department of Pharmacology and Graduate Institute of Medical Sciences, College of Medicine, Taipei Medical University, Taipei 110, Taiwan; d102092002@tmu.edu.tw (K.-H.L.); gaujang@mail2000.com.tw (K.-C.L.); d119096013@tmu.edu.tw (W.-J.L.); 2 Central Laboratory, Shin Kong Wu Ho-Su Memorial Hospital, Taipei 111, Taiwan; 3 Department of Neurology, Chi Mei Medical Center, Tainan 710, Taiwan; 4 Department of Research and Development, Institute of Ophthalmology, Joseph Eye Hospital, Tiruchirappalli 620001, Tamil Nadu, India; thomasdiagnosticcentre@gmail.com

**Keywords:** astaxanthin, LPS, Con A, mice, lymphocytes, INF-γ, IL-2, immunomodulation

## Abstract

Astaxanthin, a potent antioxidant carotenoid, plays a major role in modulating the immune response. In this study, we examined the immunomodulatory effects of astaxanthin on cytokine production in primary cultured lymphocytes both *in vitro* and *ex vivo*. Direct administration of astaxanthin (70–300 nM) did not produce cytotoxicity in lipopolysaccharide (LPS, 100 µg/ mL)- or concanavalin A (Con A, 10 µg/ mL)-activated lymphocytes, whereas astaxanthin alone at 300 nM induced proliferation of splenic lymphocytes (*p* < 0.05) *in vitro*. Although astaxanthin, alone or with Con A, had no apparent effect on interferon (INF-γ) and interleukin (IL-2) production in primary cultured lymphocytes, it enhanced LPS-induced INF-γ production. In an *ex vivo* experiment, oral administration of astaxanthin (0.28, 1.4 and 7 mg/kg/day) for 14 days did not cause alterations in the body or spleen weights of mice and also was not toxic to lymphocyte cells derived from the mice. Moreover, treatment with astaxanthin significantly increased LPS-induced lymphocyte proliferation *ex vivo* but not Con A-stimulated lymphocyte proliferation *ex vivo*. Enzyme linked immunosorbent assay (ELISA) analysis revealed that administration of astaxanthin significantly enhanced INF-γ production in response to both LPS and Con A stimulation, whereas IL-2 production increased only in response to Con A stimulation. Also, astaxanthin treatment alone significantly increased IL-2 production in lymphocytes derived from mice, but did not significantly change production of INF-γ. These findings suggest that astaxanthin modulates lymphocytic immune responses *in vitro*, and that it partly exerts its *ex vivo* immunomodulatory effects by increasing INF-γ and IL-2 production without inducing cytotoxicity.

## 1. Introduction

It is important to understand the immunomodulatory properties of herbal medicines in order to provide new insights into immune function and to uncover possible avenues for immunotherapy. Immunopharmacology aims to search for immunomodulators. Increasing evidence suggests that plant-based natural substances can serve as immunomodulators to control the outcome of certain immune responses. Herbal preparations have been shown to alter immune functions and to exert a wide range of immunomodulatory effects. Since plant-derived immunomodulatory substances increase immune responsiveness by triggering competent cells of the immune system, studies of medicinal plants are needed to substantiate claims that they possess prophylactic and therapeutic properties that are useful in the clinical setting [1].

In addition to medical properties, physiological interferon (IFN) has been reported to increase following intraduodenal administration of alimentary lectins [2]. Moreover, a study found that splenocytes of mice fed with a mixture of fish oil and high concentrations of proteins/lectins increased levels of interleukins (IL-2 and 4), and IFN-γ in response to concanavalin A (Con A) [3]. Recently, Requena *et al.* [4] also reported that oral administration of κ-casein glycopeptide, an active component of milk, upregulates the expression of tumor necrosis factor-α mRNA in Con A-stimulated splenocytes. Thus, studying the cytokine-modulating effect of medicinal herbs or foods is a useful approach to elucidating their immunopharmacological functions, mechanisms of oral immune responses to allergies, and immunotolerance.

Astaxanthin, a xanthophyll carotenoid, has been found in various microorganisms and marine animals [5]. A previous study showed that astaxanthin scavenged free radicals more effectively than did β-carotene, and inhibited lipid peroxidation more vigorously than did canthaxanthin, β-carotene or zeaxanthin [6]. Astaxanthin has been approved by the United States Food and Drug Administration (USFDA) for use as a food colorant in animal and fish feed [7]. Also, it is used as a source of pigment in the feed for salmon, trout and shrimp [5]. The consumption of astaxanthin is reported to prevent or reduce the risk of occurrence of different complaints in humans and animals [8,9]. Previous studies have shown that natural carotenoids play major roles in regulating immunity and disease etiology [10]. Dietary astaxanthin is reported to stimulate mitogen-induced lymphocyte proliferation, increase natural killer cell cytotoxicity and the delayed-type hypersensitivity response, and increase the number of total T and B cells in the peripheral blood [11]. Park *et al.* [12] showed that astaxanthin is absorbed after oral administration in domestic cats and it is subsequently utilized by blood leukocyte subcellular organelles, mostly by the mitochondria. Hitherto, research has tended to focus on the biological effects of astaxanthin, and there are no detailed reports on its immunomodulatory effects with possible molecular mechanisms. In this study, we examined the effects of astaxanthin on Con A- and lipopolysaccharide (LPS)-induced IL-2 and IFN-γ production in primary cultured lymphocyte cells *in vitro* and *ex vivo*.

## 2. Results and Discussion

### 2.1. Results

#### 2.1.1. Astaxanthin for Cell Viability and Lipopolysaccharide (LPS) and Concanavalin A (Con A) Induced Lymphocyte Proliferation

**Figure 1:**
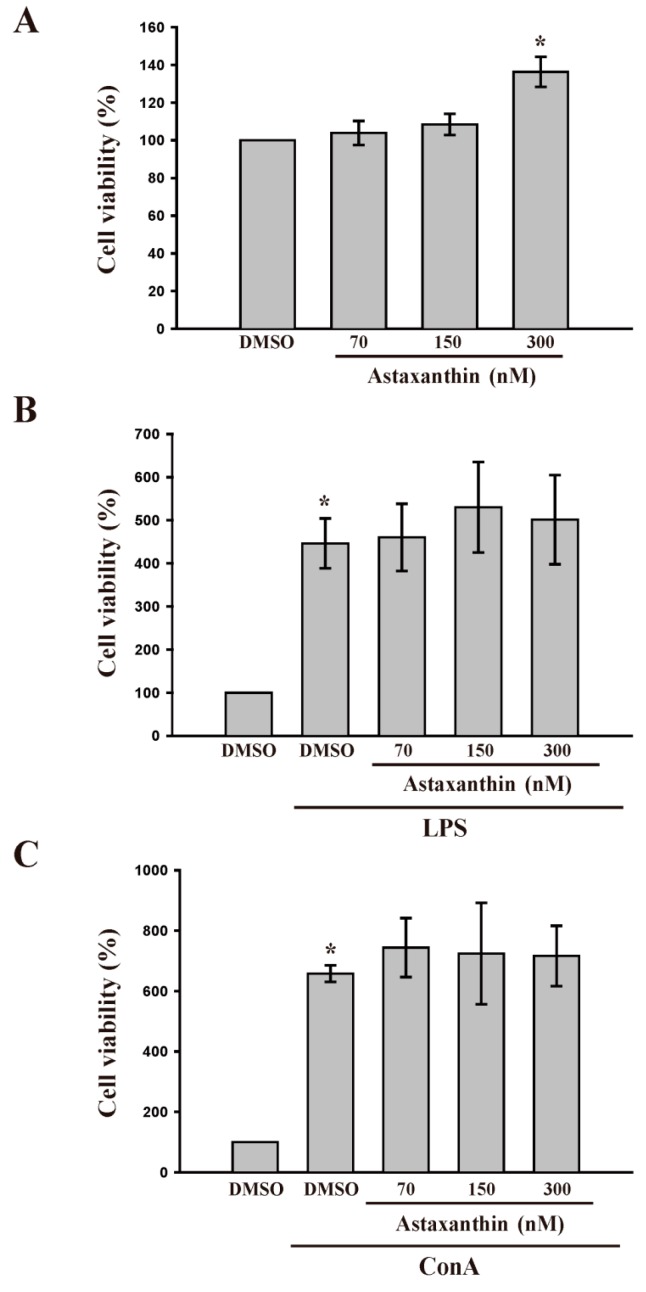
Effects of astaxanthin on cell viability in lipopolysaccharide (LPS) and concanavalin A (Con A)-activated lymphocytes. Cells were treated with astaxanthin (70–300 nM) in the absence or presence of LPS (100 μg/mL) or Con A (10 μg/mL) for 48 h. (**A**–**C**) Cell viability was determined using a 3-(4,5-dimethylthiazol-2-yl)-2,5-diphenyl tetrazolium bromide (MTT) assay (*n* = 5). Data are presented as the mean ± SD and cell viability to solvent control (DMSO) group was 100%; * *p* < 0.05 compared with solvent control (DMSO).

Cell viability and proliferation of lymphocytes was measured by conducting a 3-(4,5-dimethylthiazol-2-yl)-2,5-diphenyl tetrazolium bromide (MTT) assay. As shown in Figure 1A, astaxanthin, in concentrations of 70 and 150 nM, did not affect the viability of lymphocytes after treatment for 48 h, suggesting that astaxanthin is not cytotoxic to lymphocytes. Figure 1B,C show that treatment with LPS (100 µg/mL) or Con A (10 µg/mL) for 48 h significantly (*p* < 0.05) induced lymphocyte proliferation. However, astaxanthin, even at a high concentration of 300 nM, did not enhance this stimulation, suggesting that astaxanthin is not effective in enhancing *in vitro* LPS- or Con A-induced cell proliferation in lymphocytes.

#### 2.1.2. Astaxanthin and *in Vitro* LPS- and Con A Induced IFN-γ and IL-2 Production

IFN-γ and IL-2 are the major Th1 cytokines involved in cellular immune activation of dendritic cells and monocytes [13,14]. A previous study reported that IL-2, IL-4, IL-5 and IFN-γ production was increased in cultured murine Peyer’s patch cells *in vitro* and *ex vivo* upon non-specific T-cell stimulation with Con A [15]. Consistent with these reports, the current study shows that astaxanthin stimulated LPS-induced IFN-γ production but was not effective in stimulating Con A-induced production of IFN-γ and IL-2 *in vitro* (Table 1). In addition, in this study, astaxanthin, at a concentration of 70–300 nM without LPS or Con A stimulation, did not induce production of IFN-γ or IL-2 in lymphocytes *in vitro* (Table 1).

**Table 1:** Effects of astaxanthin on INF-γ and IL-2 production in lipopolysaccharide (LPS)- and concanavalin A (Con A)-activated primary cultured lymphocytes *in vitro*. All data are expressed as the mean ± SD (*n* = 5). * *p* < 0.05 compared with solvent control (DMSO); # *p* < 0.05 compared with LPS.

Astaxanthin Alone							LPS					Con A		
Cytokines	DMSO	Astaxanthin (nM)			DMSO	DMSO	Astaxanthin (nM)			DMSO	DMSO	Astaxanthin (nM)		
		70	150	300			70	150	300			70	150	300
INF-γ (pg/mL)	153.5 ± 2.6	153.9 ± 1.6	156.8 ± 6.4	152.5 ± 1.6	168.3 ± 29.2	840.1 ± 174.5 *	1099.9 ± 70.7 #	1037.3 ± 97.3 #	1074.8 ± 111.7 #	204.5 ± 54.0	1089.9 ± 22.9 *	1074.5 ± 12.3	1119.0 ± 9.2	1113.0 ± 7.2
IL-2 (pg/mL)	18.9 ± 6.3	17.4 ± 5.1	18.5 ± 6.7	19.6 ± 7.4	–	–	–	–	–	21.1 ± 5.8	242.0 ± 42.1 *	245.2 ± 39.9	242.2 ± 48.3	287.6 ± 32.3

**Table 2:** Effects of astaxanthin on INF-γ and IL-2 production in LPS- and Con A-activated primary cultured lymphocytes *ex vivo*. All data are expressed as the mean ± SD (*n* = 5). * *p* < 0.05 compared with solvent control (normal saline, NS); # *p* < 0.05 compared with LPS or Con A.

Astaxanthin Alone							LPS					Con A		
Cytokines	NS	Astaxanthin (mg/kg)			NS	NS	Astaxanthin (mg/kg)			NS	NS	Astaxanthin (mg/kg)		
		0.28	1.4	7			0.28	1.4	7			0.28	1.4	7
INF-γ (pg/mL)	281.2 ± 53.8	358.4 ± 70.3	455.5 ± 149.0	351.8 ± 93.4	281.2 ± 53.8	632.3 ± 172.1 *	1027.7 ± 25.2 #	865.7 ± 152.5 #	900.6 ± 195.6 #	281.2 ± 53.8	934.1 ± 181.8 *	1107.9 ± 20.2 #	1054.2 ± 20.4 #	1084.2 ± 44.5 #
IL-2 (pg/mL)	8.6 ± 6.0	37.3 ± 23.6 *	47.9 ± 11.3 *	56.9 ± 18.0 *	–	–	–	–	–	21.7 ± 6.1	187.5 ± 19.2 *	231.3 ± 34.9 #	252.0 ± 7.9 #	269.9 ± 45.6 #

#### 2.1.3. Astaxanthin and Cell Viability and LPS- and Con A-Induced Lymphocyte Proliferation *ex Vivo*

**Figure 2:**
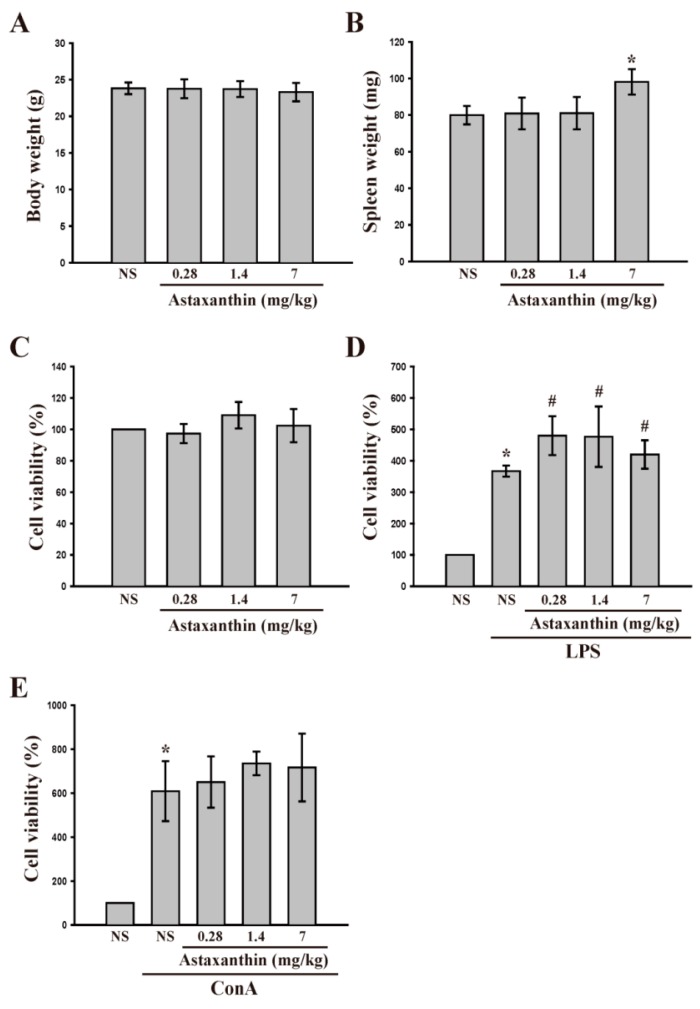
Effects of astaxanthin on cell viability in LPS- and Con A-activated lymphocytes *ex vivo*. Mice were treated orally with astaxanthin (0.28–7 mg/kg/day), or vehicle (normal saline; NS) once daily for 14 consecutive days and then the total body weight (**A**) and spleen weight (**B**) were measured. Cells were collected and cultured with or without LPS or Con A for 48 h; (**C**–**E**) Cell viability was determined using a MTT assay (*n* = 5). All data are expressed as the mean ± SD (*n* = 4) and cell viability to solvent control (NS) group was 100%. * *p* < 0.05 compared with solvent control (NS); # *p* < 0.05 compared with LPS.

Since astaxanthin exhibited differential effects on LPS- and Con A-induced production of cytokines in the cultured lymphocytes *in vitro*, we next performed *ex vivo* analysis to confirm the effect of this drug on cytokine production. Astaxanthin was injected into mice once a day for 14 consecutive days. One day after the final injection, the total body weight and the weight of the spleen were measured in each animal (Figure 2A,B). The body weight of the animals did not change. However, a high dose of astaxanthin (7 mg/kg) significantly (*p* < 0.05) increased the spleen weight. In this experiment, oral administration of astaxanthin (0.28, 1.4 and 7 mg/kg/day) apparently caused no toxicity to mice (Figure 2C) as demonstrated by the fact that the body and spleen weights (up to 0.28 and 1.4 mg/kg astaxanthin) did not change, and there was also no cytotoxicity to lymphocytes derived from mice. Moreover, astaxanthin potentiated LPS-induced cell proliferation *ex vivo* (Figure 2D), but was not effective in Con A-stimulated proliferation (Figure 2E).

#### 2.1.4. Astaxanthin and LPS- and Con A-Induced IL-2 and IFN-γ Production *ex Vivo*

Lymphocyte cells were isolated from mice and cultivated for measurement of IFN-γ and IL-2 production. In contrast to the *in vitro* study (Table 1), the production of IL-2 and IFN-γ in lymphocyte cells of mice that had been injected with astaxanthin exceeded that occurring in lymphocytes of control mice (Table 2). In addition, a significant, concentration-dependent increase of LPS- induced IFN-γ and Con A- induced IFN-γ and IL-2 production was observed in cells from astaxanthin-administered mice (Table 2).

### 2.2. Discussion

The immune system is a vital defense against tumors, cancerous growth, and infectious diseases. Immunomodulation by medicinal plants can provide additional support to conventional chemotherapy for a range of diseases, especially when selective immunosuppression is needed for autoimmune disorders. There are several diseases where immunostimulatory drugs are needed to overcome the immunosuppression induced by drugs or environmental factors, and immunosuppressants are required when there is undesired immunopotentiation. Moreover, drugs that can improve the immune system are needed to quell the immunosuppressive effects produced by stress and chronic diseases, and in situations where immune responsiveness is impaired.

Medicinal plants and their products appear to be commonly used to modulate the immune response [16]. Astaxanthin, which is found in numerous microorganisms and marine animals, showed greater immuno-modulatory effects when compared to β-carotene [17]. Dietary supplementation with astaxanthin was found to enhance antibody production and decrease humoral immune responses in aged animals [18]. A laboratory study found that astaxanthin produced immunoglobulins in human cells [19]. Although astaxanthin has diverse pharmacological activities, its immunomodulatory potential and mechanisms still remain unknown. In this study, astaxanthin, at specific doses for *in vitro* and *ex vivo* use, was found to be safe as neither cytotoxicity nor animal mortality occurred during the treatment period. Astaxanthin-treated mice did not show any change in total body weight, but spleen weights were increased at a 7 mg/kg dosage. These results are similar to those of Petri and Lundebye [20], who performed an experimental study to evaluate the organ distribution of high doses of astaxanthin in rat feed. These workers found low levels of astaxanthin in the liver, and the highest concentrations in the spleen, suggesting that the spleen is the main site of astaxanthin accumulation, where it can induce proliferation of splenic lymphocytes and increase of the spleen weight. Moreover, we present convincing experimental evidence demonstrating that astaxanthin modulates the function of immune cells *in vitro* and *ex vivo*. Our data clearly indicate that astaxanthin did not cause cytotoxicity, whereas it significantly enhanced lymphocyte proliferation even at a high concentration of 300 nM. It is broadly recognized that plant flavonoids are of profound significance for the immune deregulation diseases due to their potent immunomodulatory effects [21]. Moreover, saponins, which are active immunomodulators, are reported to stimulate specific and non-specific immunity [22]. The immunomodulatory effects of polysaccharides are widely expressed through multiple targets, such as promoting proliferation and differentiation of lymphocytes, promoting secretion of various lymphokines, and modulating the functioning of the neuroendocrine immunomodulation network [23]. In the current study, astaxanthin exerted stimulatory effects on mouse splenic lymphocyte proliferation in the presence of LPS or Con A *ex vivo*.

Cellular and inflammatory interactions are found to be associated with the progression of vascular chronic diseases. Endothelial cells alter their functions by mechanisms that involve gene expression and *de novo* protein synthesis during exposure to cytokines. The well-designed reprogramming of endothelial cells by cytokines is considered to be of great importance, especially in patients with chronic inflammation [24]. In the current study, we demonstrated that astaxanthin does not stimulate the *in vitro* production of IFN-γ and IL-2 when used alone or in Con A-stimulated primary cultured lymphocytes; however, it was found to increase IFN-γ secretion in LPS-stimulated lymphocytes. Interestingly, our *ex vivo* study revealed that oral administration of astaxanthin alone stimulated the production of IL-2 and IFN-γ in lymphocytes of mice. Also, it enhanced Con A-induced IL-2 and IFN-γ production and LPS-induced IFN-γ production. We believe that the discrepancy between these findings are mainly due to different experimental conditions and designs. Consistent with our finding, a previous study demonstrated that *N*,*N*-dimethylaminopurinepentoxycarbonyl d-arginine, increases IL-2 and IFN-γ production in human activated lymphocytic cells; the physiological relevance of this effect was illustrated by an increase of T cell proliferation [25]. IL-2 has also been shown to promote IFN-γ production in natural killer cells [26]. Oral supplementation with β-carotene in adult humans was found to increase numbers of Th and T-inducer lymphocytes [27,28]. β-Carotene supplementation was also reported to increase IL-2 and transferrin receptors in peripheral blood mononuclear cells [28,29]. Recently, it was shown that splenocytes from mice receiving β-carotene produced more IL-2 and IFN-γ than those from control mice [30]. The results of the present study suggest that administration of astaxanthin regulates the production of induced cytokines IL-2 and IFN-γ in lymphocytes *in vitro* and *ex vivo*.

## 3. Experimental Section

### 3.1. Materials

Astaxanthin was provided as a gift from Health Resource Concept., Ltd. (Taipei, Taiwan). Con A, LPS (from *E. coli* serotype O127:B8), and 3-(4,5-dimethylthiazol-2-yl)-2,5-diphenyltetrazolium bromide (MTT) were purchased from Sigma (St. Louis, MO, USA). The Mouse Interferon γ (IFN-γ) and IL-2 ELISA Ready-SET-Go kit were purchased from eBioscience (San Diego, CA, USA). Finally, the pure (≥90%) astaxanthin was dissolved in 0.5% dimethyl sulfoxide (DMSO) and stored at 4 °C until used.

### 3.2. Mice

Male BALB/c mice weighing approximately 20–25 g (6–8 weeks old), procured from BioLASCO Taiwan Co., Ltd. (Taipei, Taiwan), was used for the experiments. The mice were acclimated for 20 days prior to dosing, during which time they had free access to food and water *ad libitum*. All animal experiments and care procedures were approved by the Institutional Animal Care and Use Committee of Taipei Medical University (LAC-2013 0092; 6 November 2013).

### 3.3. Lymphocyte Preparation

**Figure 3:**
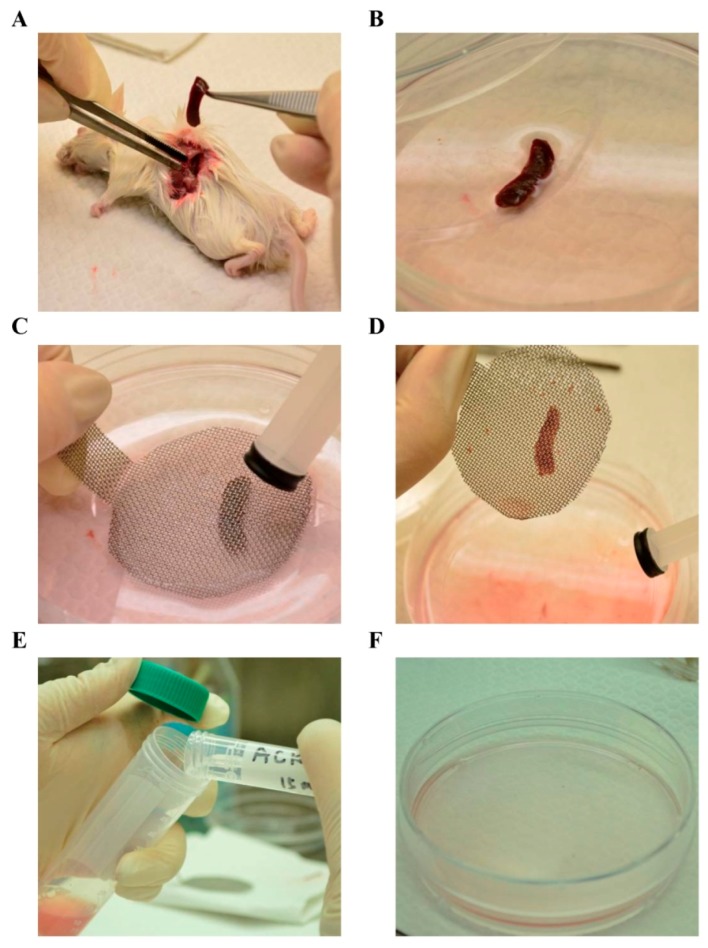
Isolation and primary cultivation of lymphocytes from spleen of BALB/c mice. (**A**) Mice were dissected and the spleen was removed using a forceps; (**B**) The spleen was directly placed in a sterile petri dish containing the Roswell Park Memorial Institute (RPMI) 1640 medium; (**C**,**D**) Single-cell suspensions were prepared by gently disrupting the spleen on a sterile wire mesh; (**E**) Red blood cells were lysed using the ACK (ammonium-chloride-potassium) lysis buffer, and centrifuged at 300× *g* for 5 min; (**F**) The lymphocyte pellets were collected and suspended with RPMI containing 5% heat-inactivated fetal bovine serum.

The spleen was aseptically removed from each mouse (Figure 3A) and placed in a sterile petri dish (Figure 3B) containing the Roswell Park Memorial Institute (RPMI) 1640 medium. Single-cell suspensions were prepared from mice spleens by disruption on sterile wire mesh (Figure 3C,D). Since lymphocyte cells are not adherent in nature, cell suspensions were centrifuged at 300 g for 5 min (Figure 3E), and the red blood cells obtained were then lysed using the ACK (ammonium-chloride-potassium) lysis buffer (15 mL) and, subsequently, 1× phosphate buffered saline (PBS; 20 mL).The lymphocyte pellets were collected through centrifugation at 300× *g* for 5 min and suspended with RPMI containing 5% heat-inactivated fetal bovine serum (Gibco, New York, NY, USA) (Figure 3F). The cell viability was determined according to trypan blue exclusion. The cells were prepared at an appropriate density depending on the scale of each experiment.

### 3.4. In Vitro Cell Viability

Cell proliferation was evaluated using a colorimetric assay. Cell viability was measured by conducting a MTT assay. In brief, cells (3 × 10^5^ cells/well) were cultured in 96-well plates and incubated with a vehicle or astaxanthin (70, 150, or 300 nM) for 48 h. MTT (5 mg/mL) was added and the cells were incubated for an additional 1 h. Since lymphocytes are suspension cells, they were centrifuged at 300× *g* for 5 min and then lysed in 400 μL of DMSO. The absorbance was measured at 570 nm by using a microplate reader. Each experiment was performed in triplicate and repeated at least five times.

### 3.5. Ex Vivo Lymphocyte Proliferation Assay

Mice received 0.28, 1.4 and 7 mg/kg/day of astaxanthin by oral gavage for 2 weeks and were then sacrificed by cervical dislocation and the spleen was removed under sterile conditions. Splenic lymphocytes were obtained as stated above (Figure 3). A total of 3 × 10^5^ lymphocytes cells were treated with or without 10 mg/mL Con A or 100 mg/mL LPS in 96-well plates for 48 h at 37 °C in a humidified atmosphere of 5% CO_2_; MTT (5 mg/mL) was added and the cells were incubated for an additional 1 h. The cells were then lysed in 400 µL of DMSO. The absorbance was measured at 570 nm by using a microplate reader. Each experiment was performed in triplicate and repeated at least five times.

### 3.6. Cytokine Evaluation

To measure cytokine production in cultured lymphocytes, culture supernatants were collected after 48 h. The levels of IL-2 and INF-γ in the supernatants were measured using the mouse IFN-γ and IL-2 ELISA Ready-SET-Go kit (eBioscience, San Diego, CA, USA) according to the manufacturer’s instructions (eBioscience). Recombinant IFN-γ and IL-2 were used to generate a standard curve, which was employed in calculating the IFN-γ and IL-2 concentrations of all samples.

### 3.7. Data Analysis

The experimental results are expressed as the mean ± SEM and are accompanied by the number of observations. For analysis of the results, a one-way analysis of variance (ANOVA) test was performed using the Sigma Stat v3.5 software (Erkrath, Germany). When group comparisons showed a significant difference, the Student-Newman-Keuls test was used. *p* < 0.05 was considered statistically significant.

## 4. Conclusions

The present study demonstrated that administration of astaxanthin modulates the production of T helper 1 cytokines, such as IL-2, as well as IFN-γ, without causing significant cytotoxic effects in primary cultured lymphocytes. In addition, astaxanthin enhanced LPS- induced immune responses by stimulating production of cytokines, as well as enhancing Con A-induced IL-2 production. Taken together, the results suggest that astaxanthin has potential value as a therapeutic or preventive agent for management of immune diseases.
